# (S)-Thienyl and (R)-Pirydyl phosphonate Derivatives Synthesized by Stereoselective Resolution of Their Racemic Mixtures With *Rhodotorula mucilaginosa* (DSM 70403) - Scaling Approaches

**DOI:** 10.3389/fchem.2020.589720

**Published:** 2020-11-11

**Authors:** Katarzyna Lubiak-Kozłowska, Małgorzata Brzezińska-Rodak, Magdalena Klimek-Ochab, Tomasz K. Olszewski, Monika Serafin-Lewańczuk, Ewa Żymańczyk-Duda

**Affiliations:** ^1^Laboratory of Biotechnology, Department of Biochemistry, Molecular Biology and Biotechnology, Faculty of Chemistry, Wrocław University of Science and Technology, Wrocław, Poland; ^2^Department of Physical and Quantum Chemistry, Faculty of Chemistry, Wrocław University of Science and Technology, Wrocław, Poland

**Keywords:** *Rhodotorula mucilaginosa*, biocatalysis, heterocyclic phosphonates, stereoselectivity, pure enantiomers

## Abstract

*Rhodotorula mucilaginosa* was successfully applied as a biocatalyst for the enantioselective resolution of the racemic mixtures of heteroatom phosphonates derivatives, resulting in receiving the following enantiomers: (S)-1-amino-1(2-thienyl)methylphosphonic acid (Product 1) and (R)-1-amino-1-(3′pirydyl) methylphosphonic acid (Product 2). Biological synthesis of both products is reported *for the first time*. Pure (S)-1-amino-1-(2-thienyl)methylphosphonic acid (Product 1) was isolated with a conversion degree of 50% after **24 h** of biotransformation was conducted on a laboratory scale under moderate conditions (1.55 mM of substrate 1, 100 mL of distilled water, 135 rpm, 25°C; Method A). The scale was enlarged to semi-preparative one, using a simplified flow-reactor (Method C; 3.10 mM of substrate 1) and immobilized biocatalyst. The product was isolated with a conversion degree of 50% just after **4 h** of biotransformation. Amino-1-(3′pirydyl)methylphosphonic acid (Substrate 2) was converted according to novel procedure, by the immobilized biocatalyst - *Rhodotorula* mucilaginosa. The process was carried out under moderate conditions (3.19 mM – substrate 2 solution; Method C1) with the application of a simplified flow reactor system, packed with the yeasts biomass entrapped in 4% agar-agar solution. Pure (R)-amino-1-(3′pirydyl)methylphosphonic (50% of conversion degree) was received within only 48 h.

## Introduction

Aminophosphonates are known as biologically active compounds (Lejczak and Kafarski, 2009). Different phosphonic derivatives interact with biological systems due to their tetrahedral geometry and the ability to donate or accept protons at physiological pH (Reda et al., 2016). Thus, such structures manifest an inhibiting activity toward aminopeptidases, antiviral (Lejczak et al., 1989; Song et al., 2003; Onita et al., 2010) and antioxidative properties, protectivity toward radiation, and also potential pesticidal features (herbicides or plant growth regulators) (Kleszczyńska et al., 2000; Kleszczyńska and Sarapuk, 2001; Sal'keeva et al., 2002; Sarapuk et al., 2003; Foss et al., 2007). There are many antiviral drugs with phosphonic motives inside the molecules, which are commercially available: e.g., cidofovir, foscarnet, and ganciclovir. Current experiments are focused on the modifications of phosphonates by introduction into their molecules' heteroatoms such as sulfur or nitrogen ones to improve the biocompatibility, bioavailability and to decrease their toxicity. Heterocyclic structures are found in nature - they built amino acids, neurotransmitters, nitrogen bases, nucleotides and vitamins (Quin and Tyrell, 2010). So, they exhibit complex biological activities, also making them important for medical chemistry (Nasir et al., 2012; El-Boraey et al., 2015). Therefore, the combination of phosphonic functionality with heterocyclic systems creates the possibility of obtaining compounds of better biological properties (Kmiecik et al., 2018). This property is strictly connected to the chiral molecules of defined absolute configuration, which can be synthesized, among others, *via* biocatalytic approach, employing flexible, fungal enzymatic systems. Thus, enzymatic systems of the *Rhodotorula* genus are commonly applied in biotechnology. As an example, PAL [Phenylalanine ammonia lyase (EC 4.3. 1.24)], responsible for the conversion of phenylalanine to *trans*-cinnamic acid and ammonia (D'Cunha et al., 1995; D'Cunha, 2005), is a base of the industrial production of *L*-phenylalanine by a microbiological method with *Rhodotorula* sp. (D'Cunha et al., 1995; D'Cunha, 2005). Also, these fungal species are known as producers of *D*-amino acid oxidases (e.g., DAAO: EC 1.4.3.3) (Gabler et al., 2000; Fantinato et al., 2001; Pollegioni et al., 2008), crucial for the production of *L*-amino acids from the racemic mixtures of the substrates *via* enantioselective, oxidative deamination of the particular enantiomers of the substrates. *D*-Amino acid oxidases are enzymes containing flavin adenine dinucleotide (FAD) and catalyzing oxidative deamination of *D*-amino acids. As a result, hydrogen peroxide and imino acid are produced, of which the latter is hydrolyzed non-enzymatically to α -keto acid and ammonia (Caligiuri et al., 2006). DAAOs are crucial in pharmacy for the production of semi-synthetic cephalosporins (Gabler et al., 2000; Pollegioni et al., 2008). Regarding the above, *Rhodotorula mucilaginosa* was applied to resolve the racemic mixtures of the phosphonates' derivatives with the sulfur or nitrogen atoms incorporated into the side functionality. These yeasts were chosen after the screening experiments showing its outstanding oxidative activity (data not shown). Employing these microbes allowed resolving the racemic mixtures of the chiral substrates during the bioconversions carried out under moderate conditions and within the short time. For the first time thienyl derivative of amino phosphonic acid was received in an optically pure form as (S)-1-amino-1-(2-thienyl)methylphosphonic acid. To our knowledge it is the first biocatalytic which resulted in the obtaining of pure enantiomer of phosphonate derivative with sulfur atom incorporated in its side functionality. The activity of *Rhodotorula mucilaginosa* was tested again toward a racemic mixture of 1-amino-1-(3′pirydyl)methylphosphonic acid in order to obtain the opposite of that previously described (Zymańczyk-Duda et al., 2019) enantiomer of this compound, and to enlarge the scale. As a consequence of inventing a new immobilization method (in 4% of agar-agar solution) (R)-enantiomer was produced.

## Materials and Methods

All chemicals were purchased from Sigma Aldrich.

### Synthesis of Racemic 1-Amino-1-(2-thienyl)methylphosphonic Acid, Substrate 1 (Figure 1)

The amino phosphonic acid was obtained in three-step synthesis based on the addition of diethyl *H*-phosphonate to the previously prepared imine and followed by acidic hydrolysis of the resulting amino phosphonate diethyl ester. According to the method described in literature (Boduszek, 1995).

**Figure 1:**
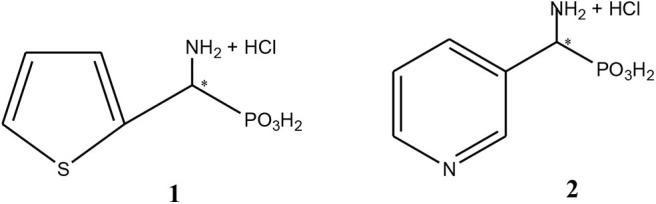
Chemical structures of 1-amino-1-(2-thienyl)methylphosphonic acid, substrate 1 and 1-amino-1-(3′pirydyl)methylphosphonic acid, substrate 2.

### Synthesis of 1-Amino-1-(3′pirydyl)methylphosphonic Acid, Substrate 2 (Figure 1)

The synthesis was carried out according to the procedure outlined in literature (Zymańczyk-Duda et al., 2019).

### Microorganism Culture Conditions

*Rhodotorula mucilaginosa* (DSM 70403) was purchased from German Collection of Microorganisms and Cell Cultures and cultivated on Potato Dextrose Broth (PDB). Fungus was cultured for 5 days in liquid medium (100 mL) on a rotary shaker (135 rpm). Then the biomass was separated by centrifugation (20°C, 5,000 rpm, 10 min), washed with water, centrifuged again, and applied for biotransformation.

### Biotransformation Procedures

#### Method A. Application of the Fresh, Wet Biomass of the *Rhodotorula mucilaginosa* for the Resolution of the Racemic Mixture of 1-Amino-1(2-thienyl)methylphosphonic Acid (Substrate 1). Laboratory (A1, A2) and Half-Preparative Scale (A3)

The wet biomass (5 g) was transferred to the 250 mL conical flask filled with distilled water (100 mL) and containing 1.55 mM of substrate **1**. Biotransformations were carried out for 5 days (135 rpm, 25°C). To track the progress of the reaction, samples were collected every 24 h. Maximum productivity (g product/g biocatalyst) was 3·10^−3^ for 50% of conversion degree, after 24 h of the process.

Depending on the outcome, preincubation of the biocatalyst in water – different starvation periods were introduced: Method A1 – 24 h; Method A2 – 48.

Method A3 - scale enlargement by multi-flaks protocol. Five gram of fresh, wet biomass portions of *Rhodotorula mucilaginosa* were placed into the 10th, 250 mL, conical flask filled with distilled water (100 mL) and containing 1.55 mM of substrate **1**, each. All flasks were incubated under the following conditions: 24h of incubation time, shaking - 135 rpm, 25°C. Then, biocatalysts were separated *via* centrifugation and water supernatants were collected, evaporated, and analyzed using NMR. Productivity of the process (g product/g biocatalyst) was 3·10^−3^.

#### Method B. Application of the Immobilized in Sodium Alginate Biomass of the *Rhodotorula mucilaginosa* (Substrate 1). Laboratory (B2) and Half-Preparative Scale (B1)

The wet biomass (30 g) was mixed with 200 mL of MOPS buffer (0.075 M, pH 7), then the suspension was added to 200 mL of 4% water solution of sodium alginate - the mixture was stirred for 5 min. The resulting suspension was added dropwise to 1,000 mL of 1% CaCl_2_ solution. The resulting capsules were left for 45 min in CaCl_2_ solution, followed by their separation through filtration on a Buchner funnel. Then the beads were placed in 500 mL 0.8% BaCl_2_ for 15 min and filtered again. The beads were then washed with distilled water, dried on a paper towel, and applied for biotransformation (Green et al., 1996).

### Method B1. Simplified Flow-Reactor (Substrate 1) - Scale Enlargement

One hundred gram of the immobilized in alginate-Ca biocatalyst (15 g of the cells) was placed in a simplified flow-reactor, that consisted of a glass column (V = 333.6 cm^3^), peristaltic pump, and conical flask. Four hundred fifty milliliter of distilled water solution of substrate **1 (3.67 mM)** was transferred to the 750 mL flask and placed on the magnetic stirrer. The flow of substrate solution through the column was forced by the peristaltic pump (5 mL/min). To control the progress of the reaction, samples (50 mL) were collected every 24 h and the reaction was carried out for 7 days (168 h).

### Method B2 - Simplified Batch Reactor (Substrate 1)

Two portions of 50 g of immobilized in alginate - Ca biocatalyst (7.5 g of the cells) were transferred to two separate Erlenmeyer flasks (500 mL), containing 100 mL of biotransformation medium each (water solution of substrate **1**- 1.55 mM). Reaction mixtures were incubated on a rotary shaker for 2 or 7 days (135 rpm, 25°C), respectively.

#### Method C (Substrate 1) and Method C1 (Substrate 2). Application of the Immobilized in 4% Agar-Agar Solution Biomass of the *Rhodotorula mucilaginosa* Supported by Polyurethane Foams. Half-Preparative Scale for Both Substrates 1 and 2 (C and C1)

#### Method C for Substrate 1

The wet biomass (50 g) was immobilized in 4% water solution of agar-agar (50 mL), then solidifying agar-agar was cut into the small irregular pieces (about 2 mm^3^). Subsequently, immobilized cells were mixed with polyurethane foams (size of pores: 1,060–1,600 μm) – 70 pieces (0.5 × 0.5 cm, Bomar Łódz, Poland). The foams were applied as a protective support for the delicate agar matrix. This mixture (in 200 mL of water) was packed into the glass column (V = 333.6 cm^3^), already filled with thin polyurethane foam layer (at the bottom) which allowed the stability of immobilized biocatalysts to be maintained under the pressure caused by flow force. Water solution of the substrate **1** (150 mL, 3.10 mM), was prepared in a separate flask (250 mL) and connected with the biocatalyst packed column, then the pump (9 mL/min) was switched on. First experiments were carried out for 4 days and monitored by sample collection (50 mL, every 24 h) and analysis. Then, after the results evaluation, the duration of the process was finally set for 4 h. Productivity of the process was observed at the level of 5.98·10^−3^ (g product/g biocatalyst).

### Method C1 for Substrate 2

Resolution of the racemic mixture (3.19 mM) of 1-amino-1-(3′pirydyl)methylphosphonic acid (**2)** was performed according to the modified Method C, with the introduction of the additional, 24 h starvation step, which was conducted as follows: after packing the column of the simplified flow reactor with biocatalyst (Method C), the pure distilled water was pumped through the system for 24 h to wash the immobilized cells. Then, the substrate solution was introduced according to the procedure described above (Method C). Productivity of the process was observed at the level 5.98·10^−3^ (g product/g biocatalyst).

*All biotransformations were carried out in triplicates*.

### NMR Analysis of the Collected Samples – Reaction Progress and Optical Purity Assignment

After the biotransformation, depending on the variant of the experiment, the biocatalyst was separated by the centrifugation (fresh, wet biomass) (20°C, 5,000 rpm, 10 min) or gravity filtration (immobilized biocatalyst). The supernatant was evaporated to dryness, and the resulting product was then examined.

#### ^31^P NMR Analysis

**Figure 2:**
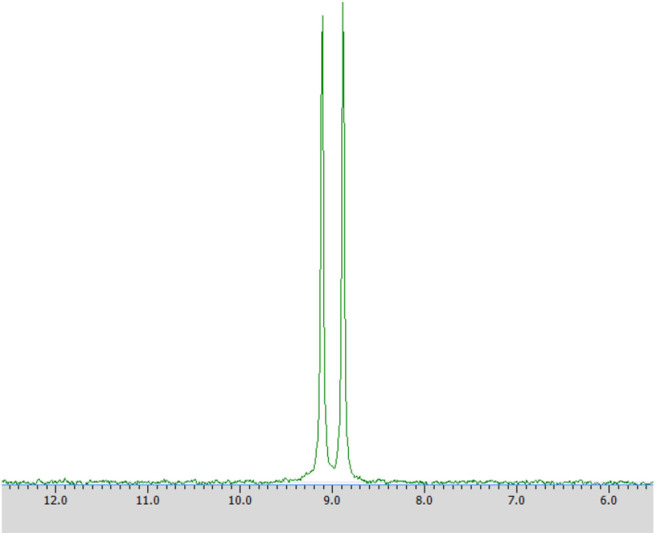
^31^P NMR spectrum of racemic 1-amino-1-(2-thienyl)methylphosphonic acid (substrate 1) with the addition of α-cyclodextrin (as chiral solvating agent) at pH 7.

**Figure 3:**
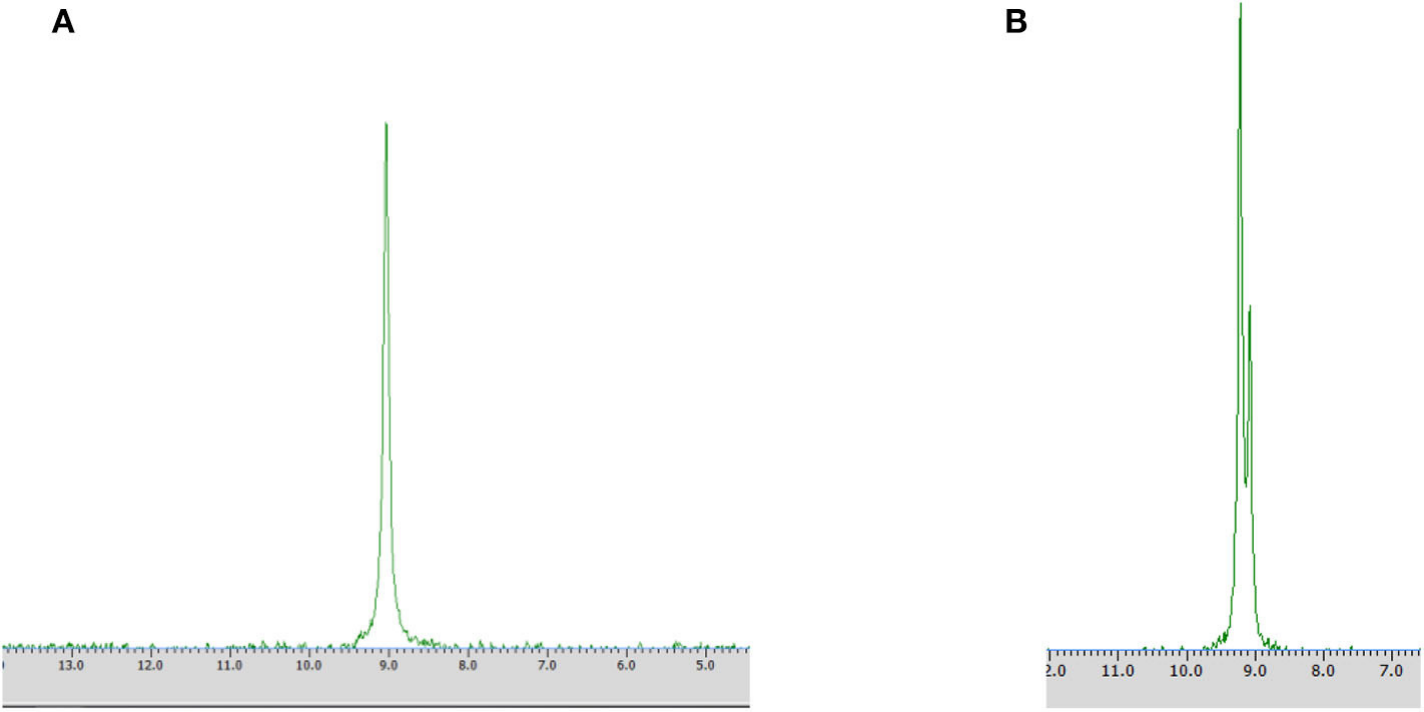
^31^P NMR spectra of biotransformation products recorded with the addition of CSA after the conversion of racemic 1-amino-1-(2-thienyl)methylphosphonic acid with *Rhodotorula mucilaginosa* (Method A) without **(A)** and with the addition of racemic mixture of substrate **1 (B)**.

Samples were prepared by dissolving in deuterium dioxide (600 mL) at the presence of α-cyclodextrin (chiral solvating agent, CSA) - to monitor the yield and stereoselectivity of the reaction (e.g., substrate **1** on Figure 2). The pH of the samples was adjusted to pH 7 for analysis of transformation of substrate **1** and to pH 11 for experiments with substrate **2** (Kmiecik et al., 2017; Zymańczyk-Duda et al., 2019). Then ^31^P NMR spectra were recorded. In the case of a 100% of the enantiomeric excess, to confirm the result, 2 mg of racemic substrate was added to the tested sample and ^31^P NMR analysis was repeated (Figures 3A,B in Results section). NMR spectra were measured on a Bruker Avance™ 600 at 243.12 MHz for ^31^P in D_2_O (99.9% of atom D). Chemical shifts (δ) were reported in ppm.

#### Absolute Configuration Assignment of (S)-1-Amino-1-(2-thienyl)methylphosphonic Acid - Product 1

Absolute configuration of the obtained, post bioconversion, (S)-1-amino-1-(2-thienyl)methylphosphonic acid was evaluated according to the literature data (Mikołajczyk et al., 2002) by the optical rotation measurement: optical rotation [α]_D_ = −14.2° (c = 2,18; 1M NaOH).

#### Absolute Configuration Assignment of (R)-1-Amino-1-(3′pirydyl)methylphosphonic Acid - Product 2

Absolute configuration was established tentatively, using the model - similar compound: 1-aminophenylmethanephosphonic acid and defined as (R)-1-amino-1-(3′-pyridyl)methylphosphonic acid (**2)** by the optical rotation measurement and according to the literature data (Kafarski et al., 1983) (optical rotation [α]_D_ = + 2.8° (c = 2.5; 1M NaOH)).

### Purification of the 1-Amino-1(2-thienyl)methylphosphonic Acid and 1-Amino-(3′pirydyl)methylphosphonic Acid by Medium Pressure Chromatography (MPLC)

After the biotransformation compound was purified by the Medium-Pressure Liquid Chromatography system: Combi Flash® Rf 150 on a reversed phase column, PuriFlash C18-HP, 15 μm, 6 g. The general procedure of purification using MPLC was carried out, as follows: 20 min of isocratic flow of pure water, 20 min from 0 to 100% of acetonitrile in water, 10 min of isocratic flow of pure acetonitrile; flow 10 mL/min, death time 1 min, R_f_ = 1.1 min. Collected fractions were analyzed by ^31^P NMR.

## Results

Application of the fresh, wet biomass of the *Rhodotorula mucilaginosa* for resolution of the racemic mixture of 1-amino-1(2-thienyl)methylphosphonic acid (substrate **1**).

### Methods A, A1, A2

Different times of biotransformation, and different biocatalyst pre-incubation periods (starvation conditions) were applied to obtain the highest enantiomeric excess of desired product. The most effective conditions were as follows: 24 h of biotransformation without biocatalyst preincubation – achieved enantiomeric excess was *e.e*. 100% (Table 1, Figure 3A) and productivity was 3·10^−3^ (g product/g biocatalyst). Optically pure product was also obtained after 48 h of biotransformation with assistance of pre-incubated cells (24 or 48 h starvation period) (Table 1). Extending the biotransformation time significantly reduced the optical purity of the obtained product as well as the overall phosphonate concentration in the samples, which indicates the prevalence of degradation processes carried out with the biocatalyst cells assistance (data no shown).

**Table 1:** Results of biotransformation of the racemic mixture of 1-amino-1(2-thienyl)methylphosphonic acid (substrate **1**) with fresh, wet biocatalysts cells.

**Method**	**Time**	***e.e*.**
A	24 h	100%
A1		95%
A2		96%
A	48 h	44%
A1		100%
A2		100%

*Conversion degrees of the processes were 50%*.

### Method A3

**Figure 4:**
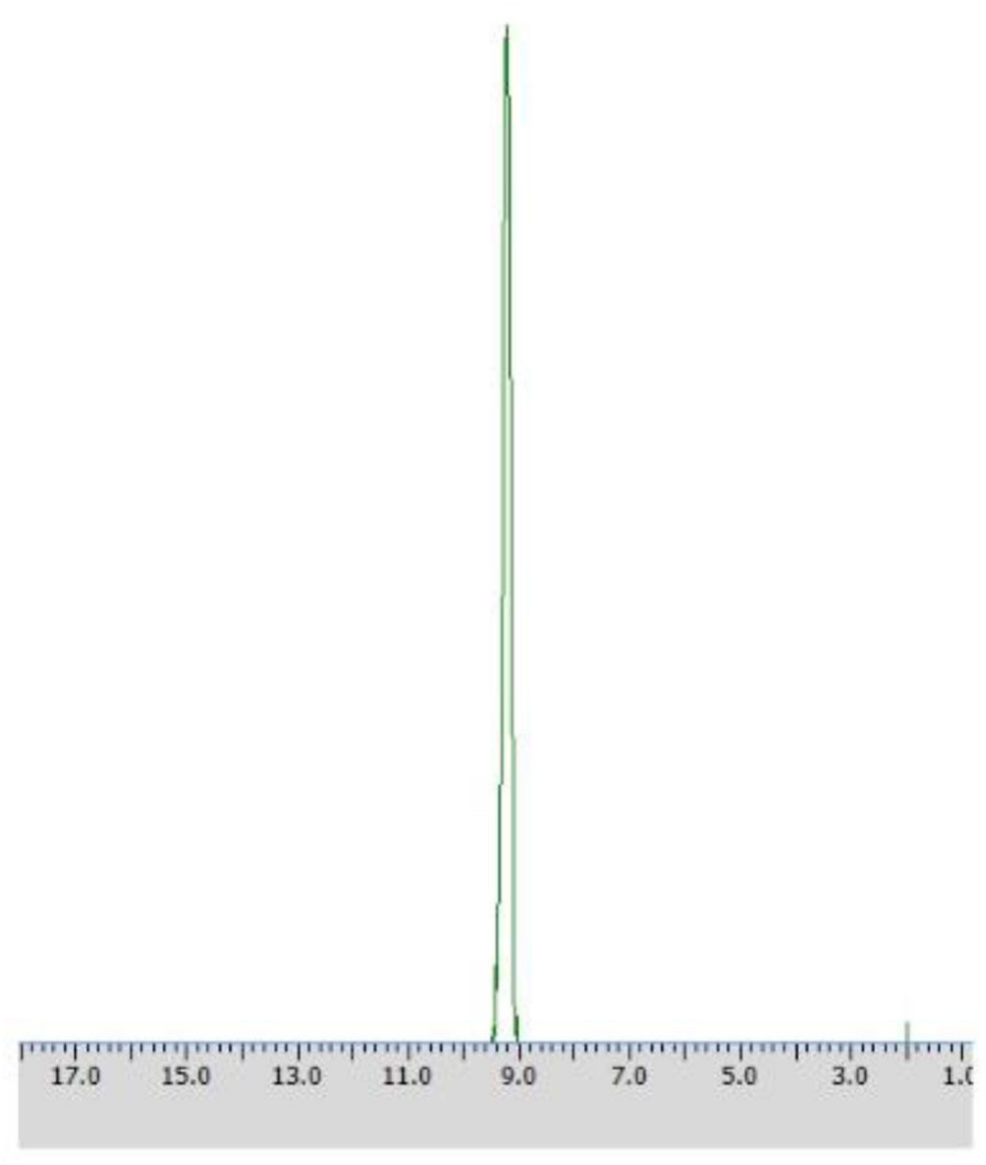
^31^P NMR spectra of post-bioconversion mixture recorded with the addition of CSA, after the biotransformation of racemic 1-amino-1-(2-thienyl)methylphosphonic acid using *Rhodotorula mucilaginosa* as biocatalyst (Method A3).

After 24 h of biotransformation, optically pure product was obtained (50% of conversion degree) as confirmed by ^31^P NMR analysis (Figure 4).

#### Application of the Immobilized Biomass of the *Rhodotorula mucilaginosa* for Resolution of the Racemic Mixture of 1-Amino-1(2-thienyl)methylphosphonic Acid (Substrate 1)

**Figure 5:**
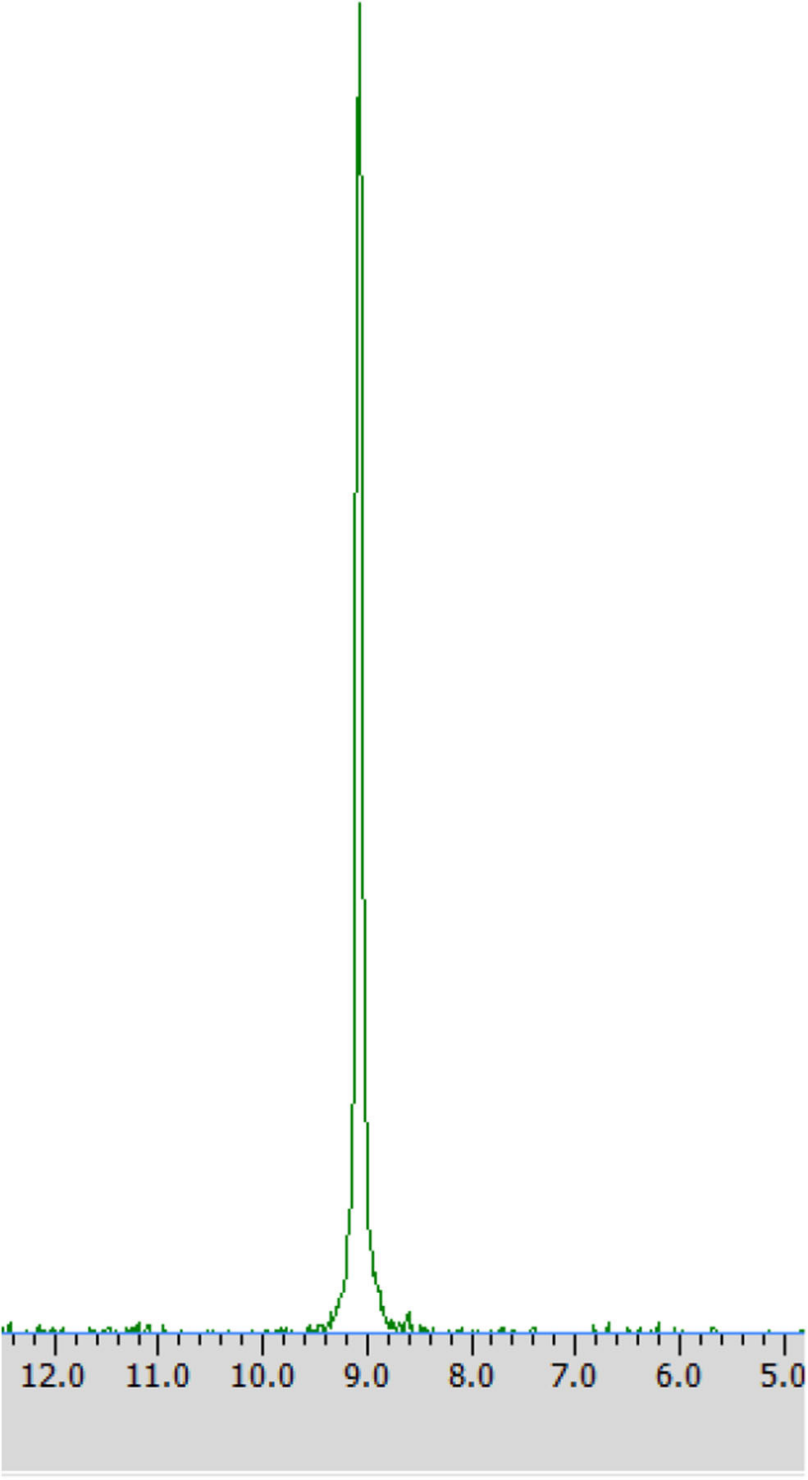
^31^P NMR spectra of post-bioconversion mixture recorded with the addition of CSA, after biotransformation of racemic 1-amino-1-(2-thienyl)methylphosphonic acid using *Rhodotorula mucilaginosa* immobilized in 4% agar-agar water solution (Method C).

In order to increase the efficiency of the biotransformation process and to decrease the sensitivity of the biocatalyst to a higher substrate concentration, immobilizations of *R. mucilaginosa* in alginate-Ca and in 4% agar-agar solution were performed. This biocatalyst was used in two simplified modes of reactors: flow and batch (the last one only for immobilization in alginate-Ca). Calcium alginate yeasts encapsulation was of low effectiveness, regardless of the type of the reactor (flow or batch). Application of flow reactor led to the products being slightly enantiomerically enriched (12–22 % *e.e*. data not shown), within 24 to 168 h. In order to improve the mass transport between encapsulated biocatalyst and the environment – a simplified model of the batch reactor with intensive mixing was applied. This positively affected the enantioselectivity of the reaction - the product was obtained with an *e.e*. of 42% after 48 h and 47% after 168 h (Table 2). Further experiments on alternative immobilization methods led to the settings of the most effective procedure (Method C) with application of agar-agar as supporting material. This time just after 4 h, entrapped in agar-agar biocatalyst, enantio selectively converted the racemic mixture of the substrate to the product with a conversion degree of 50 and 100% of *e.e*. (Table 2, Figure 5), and productivity was 5.98·10^−3^ (g product/g biocatalyst). Further time extension caused the complete degradation of the thienyl phosphonate (data not shown).

**Table 2:** Results of biotransformation of the racemic mixture of 1-amino-1(2-thienyl)methylphosphonic acid (substrate **1**) using an immobilized biocatalyst.

**Method**	**Time**	***e.e*.**
B2	48 h	42%
	168 h	47%
C	4 h	100%

*Conversion degrees of the processes were 50%*.

#### Application of the Agar-Agar Entrapped Biomass of the *Rhodotorula mucilaginosa* for Resolution of the Racemic Mixture of 1-Amino-1-(3′pirydyl)methylphosphonic Acid (Substrate 2, Method C1)

**Figure 6:**
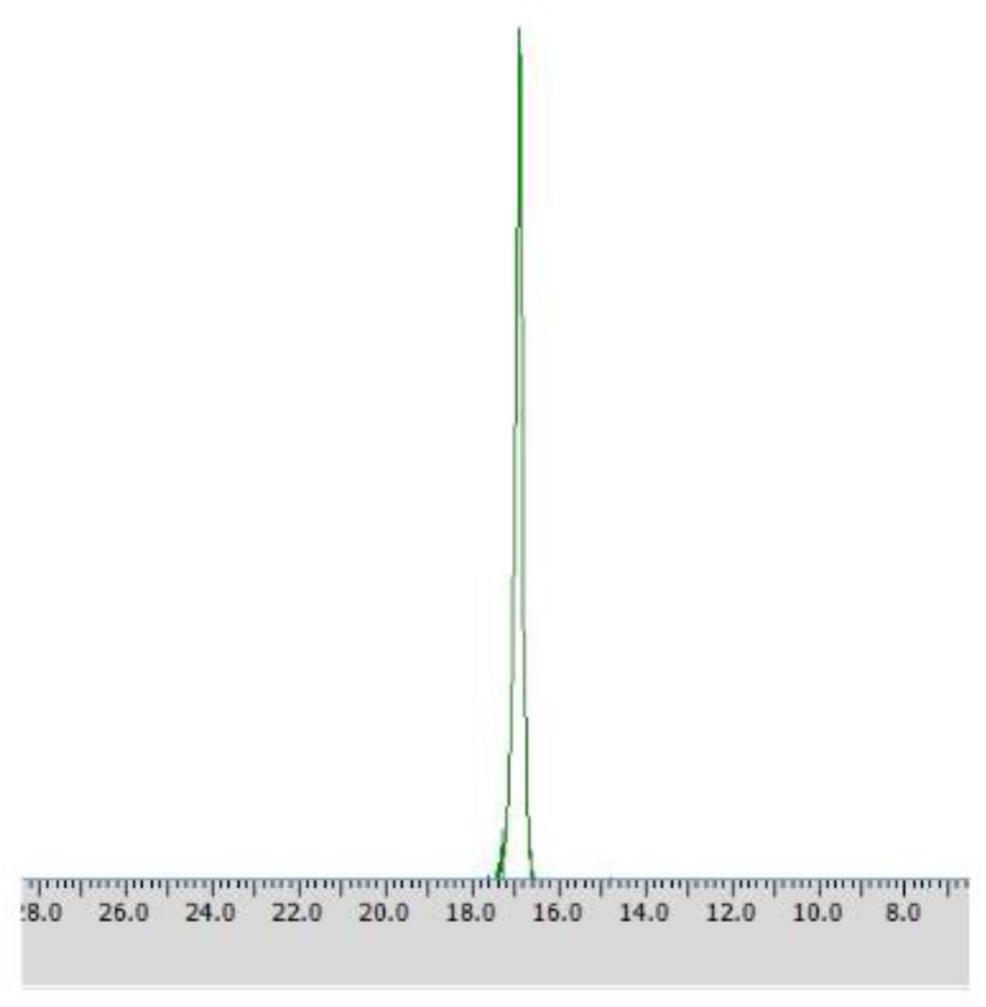
^31^P NMR spectrum of post-bioconversion mixture recorded with the addition of CSA, after the biotransformation of racemic 1-amino-1-(3′pirydyl)methylphosphonic acid using immobilized *Rhodotorula mucilaginosa* as biocatalyst (method C1).

Application of procedures described as Method C1 toward substrate **2** led to the outstanding results – pure (R) – isomer of the substrate was obtained just after 48 h with the productivity of 5.98·10^−3^ (g product/g biocatalyst), which was proved with ^31^P NMR spectra recorded in the presence of α-cyclodextrin as chiral solvating agent (CSA) (Table 3, Figure 6).

**Table 3:** Results of biotransformation of the racemic mixture of (3′pirydyl)methylphosphonic acid (substrate **2**) with immobilized biocatalyst (agar-agar entrapment).

**Method**	**Time**	***e.e*.**
C1	24 h	-
	48 h	100%
	72 h	100%
	96 h	-

“*-” means lack of the product. Conversion degrees of the processes were 50%*.

## Discussion

**Scheme 1:**
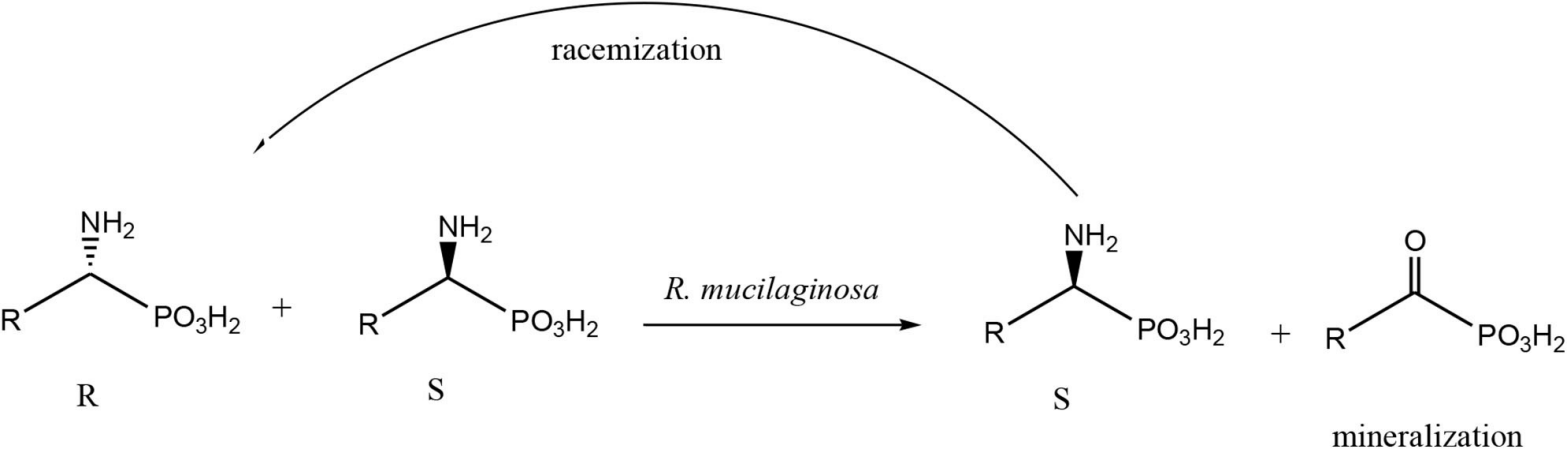
General scheme of biotransformation of amino phosphonates, applies also to substrates **1** and **2**. The path of possible racemization, a consequence of the extension of the bioconversion time is also introduced with the arrow.

Enantiomers of chiral amino phosphonic acids containing heterocyclic motifs in their structures, can be used as fine chemicals for the synthesis of biologically active compounds, mainly pharmaceuticals. The presence of pyridine or thienyl ring increases the spectrum of applications of these compounds in medical chemistry (Moonen et al., 2004; Jeught and Stevens, 2009). These compounds manifest less toxicity toward living cells than their carbon counterparts. Chemical synthesis of such derivatives in an optically pure form requires the use of expensive chemical catalysts or multi-stage syntheses, which is not cost effective at all (Saranya et al., 2019; Zhi et al., 2019). Therefore, highly selective biocatalysis is a good alternative. Microbial deracemization of chiral amino phosphonates can be achieved, among others, by deamination of one of the enantiomers and complete, further mineralization of the formed oxophosphonate (intermediate of the process), if started from the racemic mixture of the substrate (Scheme 1). Yeasts *R. mucilaginosa* were selected for such purpose due to their classification as microbes of increased activity of amino acid oxidases toward structurally different amino acids (Pollegioni et al., 2008). In order to check the enantioselectivity of the performed biotransformations, it was necessary to select the appropriate analytical method. Based upon the previous research conducted at the Department of Bioorganic Chemistry (Rudzińska et al., 2010), α-cyclodextrin (CSA) was added to the ^31^P NMR samples of the post-bioconversion mixture and this allowed for tracking of the progress, the yield and stereochemistry of the reaction. Due to the structure of this chemical auxiliary—a toroid with a hydrophobic cavity and an external hydrophilic surface—cyclodextrins are able to form guest-host inclusion complexes with hydrophobic molecules with amino phosphonates. As it is known from earlier studies, the enantiodifferentiation capacity of cyclodextrin in case of phosphonates, depends very closely on the pH values of the sample solution (Kmiecik et al., 2017). After conducting the series of experiments, pH 7 was set as appropriate for the visualization of both enantiomers of substrate **1** on the recorded ^31^P NMR spectra (Figure 2). For substrate **2** the pH value set as 11, was known from previous studies (Zymańczyk-Duda et al., 2019). *Rhodotorula mucilaginosa* was outstandingly effective toward 1-amino-1-(2-thienyl)methylphosphonic acid (substrate 1), regarding the time, conditions and the scale of the process. Already the first experiments with this substrate, carried out in accordance with method A (Table 1) gave promising results. Thus, 1 day of biotransformation resulted in the receiving of pure (S)-1-amino-1(2-thienyl)methylphosphonic acid (Figure 3A) with a conversion degree of 50%, which was verified by control analytical experiments (^31^P NMR) (Figure 3B). The mechanism of the bioconversion can be explained as follows: during the oxidative deamination of (R)-1-amino-1-(2-thienyl)methanophosphonic acid, keto phosphonate is formed as a product (Scheme 1) and then this intermediate is mineralized by viable cells, because it serves as the carbon, phosphorus, sulfur or energy source. The residues of this, biomineralized isomer are not observed on the ^31^P NMR spectra (Figure 3A). Such reaction direction moves the balance of the biotransformation into the product side, which is favorable for the increasing of the conversion degree. With the time extension, after the next 24 h of the experiment - the optical purity of the product dropped to 44% (Table 1). This is typical for the kinetic resolution of racemic substrates by biological systems and can be a consequence of the activation of the other enzymes interfering with the reaction e.g., amino acid racemases. These enzymes can transform the optically pure product: (S)-thienyl derivative, unreacted isomer, remained in the medium, again into a mixture of both enantiomers. Such racemization re-delivered the (R) – isomer, which can be again deaminated oxidatively as a part of the metabolic pathways involved in the nitrogen circulation (Scheme 1). The next days of the process, consequently carried out without feeding of the yeasts cells (in water), led to the significant decreasing of the overall concentration of the thienyl phosphonate, which was the result of the predominance of degradation processes and finally total mineralization of the tested substrate (after 3 days, data not shown). This was an important tip pointing to the time as crucial parameter for the bioconversion conducted with such an active biocatalyst. The next experiments were intended to lead to a shorter time for the bioconversion and if possible to the obtaining of the opposite isomer. These purposes can be achieved by activating the oxidases of other stereoselectivity, which are, e.g., released by biocatalyst under stress conditions introduced as, e.g., starvation pretreatment of yeasts. Biotransformations were performed with the pre-incubation - under starvation conditions carried out for 24 h (Method A1) and 48 h (Method A2). As mentioned above, such stress conditions literally affect the catalytic activity of viable cells, and as a consequence they impact the biotransformation process, forcing the yeast cells to become active toward non-physiological substrates such as phosphonates. This approach implies switching on different enzymes, particularly those involved in the secondary metabolism. This approach leads to (S)-thienyl phosphonate, which was obtained as pure enantiomer after 3 days of the whole process duration (Method A1, biotransformation period 48 h, Table 1). Also, this time, lengthening the process time was associated with the decrease in the optical purity of the product. The deficiency of nutrients causes the activation of the enzymatic systems enabling the maximum use of the available source of nutrients, in our case thienyl phosphonate. As a consequence, the process proceeds in an enantioselective manner only at the initial stage of biotransformation, when one of the enantiomers of the substrate [(R) in discussed case] is stereoselectively transformed by proper amino acid oxidase and the other one (S) remains unreacted as a product. This reaction is also a part of the cellular nitrogen circulation system because it released this element from the converted enantiomer (R). This can be a reason of further partial racemization of the obtained (S)-enantiomer to (R)-one by conversion with other types of enzymes e.g., amino acid racemase as discussed above (Scheme 1). Similar observations were recorded for the biotransformation of thienyl phosphonate (substrate **1**) carried out with the introduction of the 48 h nutrient deficit period. Reaction was completed within 2 days - optically pure product of (S) configuration was received with the 50% of conversion degree. The experimental approach with the starvation period was effective only in the case of synthesis of (S) enantiomer and was less economic, regarding the total time of the process, comparing to studies with the fresh biocatalysts, prepared according to Method A. To enlarge the process scale and to define the absolute configuration of the product of the bioconversion of the substrate **1**, half-preparative scale experiments were conducted. This allowed isolating the larger (10 times) amount of the pure (S) enantiomer of thienyl phosphonate with the productivity on the same level as in the case of Method A: 3·10^−3^ (g product/g biocatalyst). This was performed according to method A3 and as expected, an optically pure product was obtained after the 1 day of biotransformation, which was confirmed by ^31^P NMR (Figure 4) and the absolute configuration of the product **1** was determined as (S).

The results described above were the stimulation to the next set of scaling experiments. This part is always very challenging because of at least two reasons correlated to the phosphonates nature. C-P compounds are non–physiological to the living cells and they literally act as enzymes inhibitors, so these features are limitations for the applied concentrations of the starting materials. Considering this, scaling requires the immobilization of the biocatalysts to protect them against the substrate toxicity. There are no universal protocols of biocatalyst immobilization, they are established for the particular processes and are original solutions set out for phosphonates bioconversions (Zymańczyk-Duda et al., 2019). The entrapment is the most widely used technique for whole cells immobilization. This technique, in which the cells are included within a rigid network porous enough to allow the diffusion of substrates and products, protects the selected microorganism against the reaction medium (Trelles and Rivero, 2020). So, immobilized form of biocatalyst was used according to Methods B1 and C. Biotransformation carried out in a simplified flow reactor (packed-bed reactor) and with biomass entrapped in calcium alginate failed. Enantiomeric excess of products mixture increased slightly with the extension of duration of the flow and finally achieved the maximum after the 3rd day of bioconversion (*e.e*. = 22%) (data not shown). It can be assumed that this poor resolution of substrate **1** is associated with the immobilization method, which on one hand limited the mass transport between the biocatalyst and the environment and on the other hand, with time extension, allowed for the escape of the cells from the supporting capsules. This was the reason for the invention of the other immobilization procedure, strictly developed for the applied yeasts and based upon the biomass immobilization in agar-polymeric network (Method C). This time, after 4 h of biotransformation, the enantiomeric excess of the product was 100% (Figure 5) with the 50% of conversion degree and the productivity was doubled, compared with the results obtained according to Method A. The entrapment of the fungal biocatalyst in agar-agar polymeric network allowed the contact of the biomass with the substrate solution to be increased. Also, this time, the extension of biotransformation time resulted in re-racemization of the product, according to mechanisms discussed previously in the text (Scheme 1). The efforts of scaling were developed also, as mentioned above, with the simplified batch reactor and with the cells immobilized in calcium alginate for the 1.55 mM of solution of substrate **1** (Method B2), counting on the gentle shaking to improve the mass exchange between the biocatalysts and the reaction medium and limit the escaping of the cells from the capsules. Under these conditions, the resulting resolution achieved the 47% of *e.e*. only (Table 2). Experiments conducted with *Rhodotorula mucilaginosa* proved its outstanding activity toward phosphonate **1** (thienyl derivative) and were the reason of returning to the previously described experiments with the 1-amino-1-(3′pirydyl)methylphosphonic acid (substrate **2**) (Zymańczyk-Duda et al., 2019). Applying of method C1, after just 2 days of biotransformation, the resolution of the racemic mixture of 1-amino-1-(3′pirydyl)methylphosphonic acid was accomplished and pure (R) isomer was received with 50% of conversion degree (Figure 6). This result was really surprising, when compared to our previous studies that resulted in the optically pure (S)-1-amino-1-(3′pirydyl)methylphosphonic acid formation, also with *Rhodotorula mucilaginosa* assistance. The difference between these two approaches was in the achieving of the process biocatalysts readiness and as a consequence the enzymes of opposite stereoselectivities manifested their activity. Among others it has to be stressed, that fungal enzymatic systems are really extraordinary, considering their activities (also toward the xenobiotics) and selectivity. It is known from the literature data that the particular immobilization procedures influence the activity of the membrane-linked enzymes, sometimes leading to their complete deactivation (Csuk and Glaenzer, 1991). Previous experiments [resulted in (S) enantiomer – from substrate **2**] employed two different types (molds and yeasts) and forms of biocatalysts: fresh yeasts *Rhodotorula mucilaginosa* cells and immobilized *Penicillium funiculosum* mycelium. Thus, comparing the previous and current results of substrate **2** conversion by yeasts, not a single enzyme but different enzymes of different stereoselectivities are responsible for the reaction: located in the fresh, non-intact by any external factors cells and in the second case located in the polymeric matrix. Such supports affect the activities of membrane-linked proteins—in discussed work - enzyme of (R)—preferences, what allowed manifesting of the activities of (S)—specific enzymes (probably of low enantioselectivity E), thus this enantiomer is converted and enantiomer (R) remains unreacted. Therefore, different biocatalyst pretreatments can lead to the opposite enantiomers (Csuk and Glaenzer, 1991).

## Conclusions

The outstanding activity of *Rhodotorula mucilaginosa* toward thienyl and pyridyl derivatives of phosphonates allowed for the achieving of the enantioselective resolution of their racemic mixtures, which resulted in obtaining (S) and (R) enantiomers, respectively on a laboratory and half-preparative scale. This is particularly important in the case of compound **2** and regarding the previously conducted experiments (Zymańczyk-Duda et al., 2019), because the application of one biocatalyst under different conditions, led to both optical isomers of pyridyl phosphonate.

## Data Availability Statement

The raw data supporting the conclusions of this article will be made available by the authors, without undue reservation.

## Author Contributions

MB-R and EŻ-D conceived the idea and supervised the project. TKO was responsible for the substrates' synthesis. MB-R, MS-L, KL-K, and TKO were responsible for the experimental part of work. MB-R, EŻ-D, and MK-O wrote the manuscript. All authors contributed to the article and approved the submitted version.

## Conflict of Interest

The authors declare that the research was conducted in the absence of any commercial or financial relationships that could be construed as a potential conflict of interest.
